# Characterizing and Modeling Breathing Dynamics: Flow Rate, Rhythm, Period, and Frequency

**DOI:** 10.3389/fphys.2021.772295

**Published:** 2022-02-21

**Authors:** Nicholas J. Napoli, Victoria R. Rodrigues, Paul W. Davenport

**Affiliations:** ^1^Department of Electrical and Computer Engineering, University of Florida, Gainesville, FL, United States; ^2^Human Informatics and Predictive Performance Optimization (HIPPO) Lab, University of Florida, Gainesville, FL, United States; ^3^Breathing Research and Therapeutics (BREATHE) Center, University of Florida, Gainesville, FL, United States; ^4^Department of Physiological Sciences, University of Florida, Gainesville, FL, United States

**Keywords:** breathing dynamics, instantaneous flow, work of breathing, breathing frequency, breathing rate, breathing period, breathing rhythm, compensation

## Abstract

The characterization of breathing dynamics provides researchers and clinicians the ability to differentiate respiratory compensation, impairment, disease progression, ventilator assistance, and the onset of respiratory failure. However, within many sub-fields of respiratory physiology, we still have challenges identifying changes within the breathing dynamics and critical respiratory states. We discuss one fundamental modeling of breathing and how modeling imprecise assumptions decades ago regarding breathing are still propagating into our quantitative analysis today, limiting our characterization and modeling of breathing. The assumption that breathing is a continuous sinusoidal wave that can consist of a single frequency which is composed of a stationary time-invariant process has limited our expanded discussion of breathing dynamics, modeling, functional testings, and metrics. Therefore, we address major misnomers regarding breathing dynamics, specifically rate, rhythm, frequency, and period. We demonstrate how these misnomers impact the characterization and modeling through the force equations that are linked to the Work of Breathing (WoB) and our interpretation of breathing dynamics through the fundamental models and create possible erroneous evaluations of work of breathing. This discussion and simplified non-periodic WoB models ultimately sets the foundation for improved quantitative approaches needed to further our understanding of breathing dynamics, compensation, and adaptation.

## 1. Introduction

Capturing and characterizing breathing dynamics (Mortola et al., 1982) has a wide range of pivotal applications, such as differentiating the progression of respiratory diseases (Scano et al., 2010), differentiating the type of mechanical ventilation needed for a patient (Banner et al., 1994; Wilson et al., 1999), predicting extubation outcome (Teixeira et al., 2009), mask design (Tian et al., 2020), and aeromedical applications (Grönkvist et al., 2008; Ludwig et al., 2019). The breathing dynamics of pressure, volume, and airflow are all linked to the forces generated with breathing, but their analysis is not always sensitive for discriminating breathing changes within diseases and other impactful environments to breathing (Kallet et al., 2005; Johnson and Mitchell, 2013). Within neuromuscular diseases, this lack of discrimination of breathing dynamics impacts the prognostication techniques for detecting a rapid failure of breathing and causes patients to unexpectedly reach their limit for breathing compensation leading to ventilatory failure, referred to as “falling off the cliff” (Johnson and Mitchell, 2013). These issues of analysis for breathing mechanics and metrics of respiratory performance are even seen within ventilator weaning (Tobin et al., 1986; Teulier et al., 2013; Matecki et al., 2016), aerospace (Grönkvist et al., 2008), tissue engineering (Huang et al., 2021), and exercise (Younes and Kivinen, 1984; Dominelli and Sheel, 2012). Thus, there is something innately imperfect with the way we are analyzing, assessing, and hypothesizing about breathing mechanics and their dynamics. This brings into question if we really are assessing the full dynamic range of the patients breathing capacity and applying the appropriate analysis.

Therefore, it is of supreme importance to have at our disposal efficient metrics and techniques that can better discriminate the onset of respiratory failure, the progression of respiratory diseases, and ventilatory adaptation. Out of the many breathing variables to characterize, instantaneous airflow has great potential to uniquely assess breathing dynamics. Instantaneous airflow has been advocated to explain patterns of neural stimulation of respiratory muscles (Milic-Emili and Zin, 2011), and the muscular, elastic, flow-resistive, and inertial forces of breathing (Gray and Grodin, 1951; Milic-Emili and Zin, 2011). However, no solution has been generated to decompose instantaneous airflow into a meaningful model that enables interpretation and prediction (Gray and Grodin, 1951; Milic-Emili and Zin, 2011). Thus, analysis of instantaneous airflow can provide a great deal of information if the properly advanced analysis is utilized, providing powerful diagnostic biomarkers within the respiratory field.

Based on foundational instantaneous airflow models for breathing that estimate elastic, turbulent, and viscous forces which utilize differential equations of a sinusoidal wave with a single fixed frequency (Otis et al., 1950; Gray and Grodin, 1951), we demonstrate the need to improve our terminology that characterizes breathing waveforms, more advanced techniques of time-frequency analysis, and provide an updated Work of Breathing (WoB) model for non-periodic breathing filling in the missing links to fully characterizing breathing mechanics.

## 2. Current Airflow Methods for Modeling Breathing

Prior modeling using sinusoidal waveforms must be modified to account for real-time airflow patterns that have a more complex waveform. Thus, the transition from pure sinusoidal airflow to the non-sinusoidal waveforms pattern of breathing commonly found in normal breathing is necessary. We provide the nascent foundational respiratory modeling that shaped our current understanding of the mechanics of breathing, and defines the metrics we use to characterize breathing. We identify how these nascent models, although appropriate for the initial analytical model, have assumptions which we now know can be enhanced by the current advances within the field of signal analysis and mathematics. We aim to address these imprecise assumptions that have perpetuated throughout the years in order to demonstrate the need for more advanced breathing dynamics modeling.

### 2.1. Foundational Flow Model for Breathing Dynamics

The foundational work for understanding breathing dynamics was generated in the late 1940s and early 1950s, which created generalized equations of the forces related to breathing dynamics and WoB (Fenn et al., 1946; Otis et al., 1950; Otis, 1954; Abboud et al., 1986; Bachy et al., 1986; Benchetrit et al., 1989). These fundamental papers have impacted the science of breathing for decades with minimal changes over time. WoB is a representation of the amount of energy required to overcome the elastic and resistive elements of the respiratory system that move gas into and out of the lung during breathing (Otis et al., 1950; Stoller and Hill, 2012). To calculate the WoB that was formally modeled and still used today requires the measurement of pleural pressure. This usually requires an esophageal balloon to be placed within the subject to measure the work. However, in the majority of the cases, a spirometer and a pneumotachograph are used to assess breathing mechanics and patterns, measuring only the pressure and airflow at the mouth. Since “total work” requires the pleural pressure measurement, in most settings, we measure only “added work.” Going beyond our classical definition of WoB breathing (added work), $W=\overset{t=T}{\underset{t=0}{\int }}P\left(t\right)V\left(t\right)$, we can better estimate “total work” for a breathing cycle without pleural pressure measurement through the instantaneous flow velocity patterns using a pneumotachogram (Otis et al., 1950).

**Figure 1:**
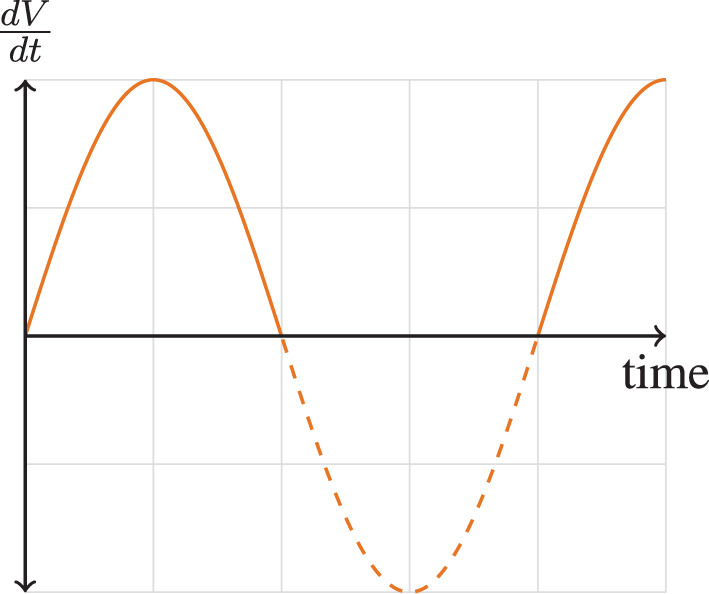
Flow velocity pattern.

By understanding this foundational instantaneous flow velocity and volume model, we demonstrate the misconceptions and key features that are neglected in the analysis of breathing because of the elegant but simplified modeling assumptions made 70 years ago. This velocity and volume model for the “total” force required for breathing accounts for elastic forces (*K*), viscous resistance (*K*′), and turbulent resistance (*K*″), and it defines the total force required *F*_*T*_, by


(1) $$\begin{array}{r} F_{T}=KV+K^{\prime }\left(\frac{dV}{dt}\right)+K^{\prime \prime }{\left(\frac{dV}{dt}\right)}^{2} \end{array}$$

where V is the volume of air and $\frac{dV}{dt}$ is the change of volume of air over time. This method that defined breathing dynamics used sinusoidal wave of inspiration,


(2) $$\begin{array}{r} \frac{dV}{dt}=a\cdot sin\left(bt\right) \end{array}$$

where $\frac{dV}{dt}$ is the velocity of airflow, a is the maximal velocity, and $\frac{b}{2\pi }=f$ is the frequency of breathing. This velocity pattern in Equation 3 can be visually shown in Figure 1. This allows us to define the tidal volume, *V*_*T*_, as


(3) $$\begin{array}{r} \;V_{T}=\int_{0}^{\frac{\pi }{b}}a\cdot sin\left(bt\right)dt=\frac{2a}{b}=\frac{a}{\pi f}. \end{array}$$

The differential equation for work (*dW* = *PdV*) can be created by using Equation 1 and $\frac{dV}{dt}=asin\left(bt\right)$,


(4) $$\begin{array}{r} dW=KV+K^{\prime }a^{2}sin^{2}\left(bt\right)dt+K^{\prime \prime }a^{3}sin^{3}\left(bt\right)dt \end{array}$$

where this expression can be thought of as three components, the modeling of the elastic work, viscous work, and the work associated with turbulent resistance, respectively. The integration of the Equation 4, produces the equation of work for a single inspired breath using the airflow velocity data, defined by


(5) $$\begin{array}{r} W=\frac{1}{2}KV_{T}^{2}+\frac{1}{4}K^{\prime }\pi^{2}fV_{T}^{2}+\frac{2}{3}K^{\prime \prime }\pi^{2}f^{2}V_{T}^{3}. \end{array}$$

This model serves its purpose perfectly when estimating the forces of an “ideal” breath, that is, a breath that has only one frequency, and only for a single breath. The first term of Equation 5, the elastic force, has no direct contribution from the frequency of the instantaneous flow. However, frequency does significantly impacts both viscous (resistive) and turbulent forces. Note that as the instantaneous flow frequency increases, the total amount of work exponentially increases due to the viscous and turbulent elements. Likewise, the elastic work drops as a contributing factor from the total work as frequency increases. Thus, we can see the instantaneous flow frequency is incredibly important and can show exponential changes in forces within the viscous and turbulent forces. However, regardless of the magnitude of these higher frequency components, neglecting them in the analysis would prevent researchers and clinicians from characterizing key factors that could be impairing breathing (e.g., obstructed breathing and etc.). Thus, capturing the accurate and precise frequency content of a breath is paramount.

#### 2.1.1. Understanding the Variable Called “Frequency”

The frequency variable that is defined in these breathing models is modeled as a pure sinusoidal wave, which was based on elastic force models and was expanded to capture the turbulent and viscous components as well. The most simple model can be viewed as a spring equation (Hooke's law), *F* = *kx*, where F is the force, k is the spring constant, and *x* is the distance of displacement (e.g., the lungs expanding). This spring constant, *k*, is a function of frequency, *f*, where, *k* = *mω*^2^ = *m*(2π*f*)^2^. The frequency describes the speed of how the spring displaces the mass, causing an oscillating force, where an “ideal” spring continues to oscillate and never attenuates. Likewise, the airflow velocity of a breath is described by this frequency. However, unlike an ideal spring that would continue oscillating, the exhalation stage of the breath does not continue the same harmonic (frequency). Our breath is paused or slowed down during the expiration stage of the breath, altering our rhythm of breathing. Further, unlike an ideal spring that has one frequency (a single harmonic), a single breath is linked to the sum output of all our respiratory muscles acting together to alter our chest cavity and to generate the forces for our breath. Thus, multiple frequency flow components within a single breath can be generated.

#### 2.1.2. “Frequency” and the Model Impact

Since these models assume sinusoidal breathing, this has impacted how we have interpreted frequency within breathing and how these models translated to real breathing dynamics since we simply do not breathe in a sinusoidal fashion. Each inspired and expired breath are not equivalent in their shape and timing. The presented model serves its purpose perfectly when estimating the forces for a single “ideal” breath, that is, a breath that has only one frequency, and only for a single breath. Thus, under perhaps special conditions, such a model can give an acceptable approximation of the forces linked to a single breath and potentially over a series of breaths. Moreover, even if these equations' final results served only as an approximation, they are leaving out potentially critical information characterizing a breath. However, these model approximations breakdown when the shape of the breath contains multiple frequency components, when the instantaneous frequency does not match the breathing rate, and when a person compensates during breathing causing the shape of the waveform to change over each breath. Thus, when we utilize descriptive characteristics such as mean inspiratory airflow, tidal volume, rate of breathing (breaths/per minute), breathing period, and other metrics to characterize breathing, we have to better understand what information may be missing or ineffectively evaluated. This missing and imprecise information that characterizes breathing can potentially provide the next steps in understanding breathing and differentiating respiratory states. Thus, these modeling assumptions have created misnomers on our interpretation and characterizing of breathing because of this term “frequency.”

## 3. Characterizing Breathing Flow Patterns

In this section, due to simplified sinusoidal modeling, we demonstrate how the misnomer of the “true” variable of frequency is actually never appropriately captured to fully characterize an individual's breathing patterns. We demonstrate that sometimes minuscule or even large factors of frequency information can be overlooked and not utilized in the analysis of differentiating respiratory compensation, impairment, disease types, ventilator assistance, and the onset of respiratory failure. Thus, we are leaving out respiratory information that potentially characterizes these various cohorts within the breath dynamics. Our focus on this modeling misnomer highlights the differences between rate, rhythm, period, and frequency of breathing.

### 3.1. Decomposing Breathing Rate

The sinusoidal breathing models, when extrapolated over a minute period or longer, are referred to as breathing rate and often interchanged with the term breathing frequency (Evans et al., 1999; Kuipers et al., 2003; Kallet and Diaz, 2009; Tipton et al., 2017; Tams et al., 2020; Vermeulen et al., 2020). Breathing Rate (*B*_*Rate*_) is typically defined as the number of breaths in a minute (breaths per minute, BPM),


(6) $$\begin{array}{r} B_{Rate}\left(BPM\right)=\frac{1}{T}\cdot 60=f\cdot 60, \end{array}$$

where T is the period of the breath and f is frequency. However, this should not be considered the frequency of breathing when we discuss realistic breathing signals since these are two different characteristics of breathing and are only equivalent for a special case. For a sinusoidal breathing model, we can define the airflow pattern, $F_{sin}$, as,


(7) $$\begin{array}{r} F_{sin}\left(k\right)=a\cdot sin\left(2\pi ft\left(k\right)\right) \end{array}$$

where a is the amplitude of the airflow, f is the frequency of the airflow and t is the time duration. Through the segmentation of the stages of a breath (inspiration and expiration) for sinusoidal breathing, we can further define frequency as,


(8) $$\begin{array}{r} \;f=\frac{1}{T_{Tot}}=\frac{1}{T_{I}+T_{E}}=\frac{1}{2\cdot T_{E}}=\frac{1}{2\cdot T_{I}} \end{array}$$

where *T*_*I*_ is the time for the inspired breath, *T*_*E*_ is the time for expired breathing, and *T*_*Tot*_ is the full period of the inspired and expired time of one cycle of a breath. This ideal sinusoidal exemplar where $\frac{1}{2\cdot T_{I}}=\frac{1}{2\cdot T_{E}}$ (simplistically, *T*_*I*_ = *T*_*E*_) is the only case where the terms “frequency” and “rate” are equivalent and would be interchangeable. However, it is rarely observed in real breathing dynamics that our time of inspiration is equivalent to the time of expiration, where many investigators report both characteristics (Clark and Von Euler, 1972; Sun and Liu, 2009), *T*_*I*_ ≠ *T*_*E*_, which means our signal is not sinusoidal. Thus, when the overwhelming majority of breathing occurs for non-sinusoidal continuous waveforms, the previous equation does not hold,


(9) $$\begin{array}{r} f\neq \frac{1}{T_{Tot}}=\frac{1}{T_{I}+T_{E}}\neq \frac{1}{2\cdot T_{E}}\neq \frac{1}{2\cdot T_{I}}, \end{array}$$

when *T*_*I*_ ≠ *T*_*E*_. Therefore, breathing rate cannot refer to the frequency content of the breath, making Equation 6 simply false. Since these fundamental models use the variable breathing frequency to describe the work and the breathing forces, and frequency is not equal to breathing rate, the paramount point is that breathing rate is not fully characterizing the work and forces that are being produced during breathing. However, breathing rate is still a very important variable to characterize breathing but does not capture the full picture of a breathing state.

**Figure 2:**
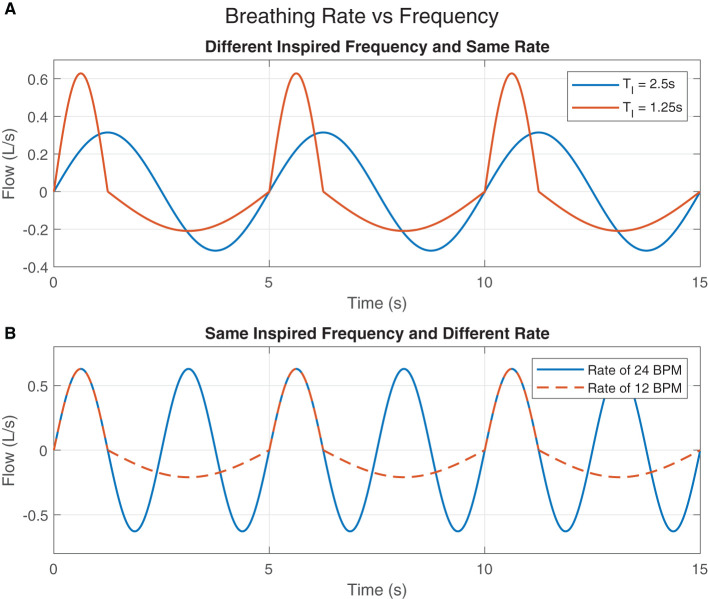
Breathing Rate vs. Frequency: **(A)** The inspired breaths' frequencies are different but the rates of breathing are the same. **(B)** The rates of breathing are different but the inspired breaths' frequencies are the same.

Figure 2A shows two idealized flow patterns to represent breathing. The blue one has a *T*_*I*_ of 2.5 s while the other one has a *T*_*I*_ of 1.25 s. However, both of them have a *T*_*Tot*_ of 5 s. Therefore, even with different inspired frequencies, both breathing patterns have the same breathing rate of 12 BPM. Figure 2B also shows two idealized flow patterns to represent breathing. The blue one has a *T*_*Tot*_ of 2.5 s, where both *T*_*I*_ and *T*_*E*_ are 1.25 s. Because this pattern is continuous and repeats over time, we consider this to have a rate of 24 BPM. The orange one also has a *T*_*I*_ of 1.25 s, however *T*_*E*_ is 3.75 s, resulting in a *T*_*Tot*_ of 5 s. Thus, the rate of breathing of this flow pattern is 12 BPM.

### 3.2. Decomposing Breathing Period

In order to characterize realistic breathing, which is a time-variant and non-sinusoidal, researchers have used half of the breathing cycle or half of the period, *T*_*I*_ and *T*_*E*_ to calculate an “instantaneous” frequency of an inspired breath, $f_{I}=\frac{1}{2\cdot T_{I}}$ or expired breath, $f_{E}=\frac{1}{2\cdot T_{E}}$ (Mortola, 2019; Hof, 2021). This method of characterizing breathing is often used to explain breathing but still inappropriate to be considered the term frequency that we know of in these fundamental breathing models. As discussed in Section 2.1.1, a single inspired breath is linked to the sum output of multiple respiratory muscles acting together to alter our chest cavity and to generate the forces for our breath. Thus, multiple frequencies are actually formed by instantaneous airflow signals for the inspired and expired stages of a breath.

We present a modeling approach of how frequency impacts the period of inhalation. We consider impulses of sub-components of the inhalation breath, which are each composed of a single breathing frequency. This impulse approach mimics how multiple respiratory muscles would impact the increase of airflow, similar to Equation 3 but with multiple waveforms. This is based on the assumption that respiratory muscles would likely impact the airflow like an impulse. By approaching the problem this way, we demonstrate that *T*_*I*_ actually does not calculate the true “instantaneous” frequency but rather can potentially underestimate the calculation.

#### 3.2.1. Analysis of Sub-components as Single Pulses

Let us consider a breath's inhalation stage is composed of multiple “instantaneous frequencies,” *f*_*I*_*i*__, where *i* = [1…*N*], denotes the number of sub-components associated to a specific frequency. Building off the fundamental flow models presented in Section 2, we create multiple flow models associated with various frequencies,


(10) $$\begin{array}{r} F_{sin_{1}}\left(k\right)=a_{1}\cdot sin\left(2\pi f_{1}t_{1}\left(k\right)\right),\\ F_{sin_{2}}\left(k\right)=a_{2}\cdot sin\left(2\pi f_{2}t_{2}\left(k\right)\right),\\ \vdots \\ F_{sin_{N}}\left(k\right)=a_{N}\cdot sin\left(2\pi f_{N}t_{N}\left(k\right)\right), \end{array}$$

where the sequence *t*_*i*_(*k*) is constrained by $k=\left[0,1,\ldots \frac{1}{2\cdot f_{i}}\right]$. This constraint within *t*_*i*_(*k*) creates a single impulse for each sinusoidal that is bounded by its own half of a period, which stops at the zero-crossing. We will assume each frequency sub-component to be independent of each other with a single pulse during the inhalation stage of the breath with the same phase information (i.e., the same starting point of the sinusoidal). This allows us to assume that the breathing forces from sets of respiratory muscles contract in unison as a single one-time effort to produce a total amount of airflow during inspiration, $F_{sin_{T}}\left(k\right)$, where


(11) $$\begin{array}{r} F_{sin_{T}}\left(k\right)=F_{sin_{1}}\left(k\right)+F_{sin_{2}}\left(k\right)+\ldots +F_{sin_{N}}\left(k\right). \end{array}$$

This produces a single impulse of breathing, $F_{sin_{i}}\left(k\right)$, with its own *f*_*I*_*i*__ and *T*_*I*_*i*__ during the inhalation stage of the breath. Through the relationship of frequency and period, consider $F_{sin_{T}}\left(k\right)$ that is composed of four impulses of breathing (N = 4), where


(12) $$\begin{array}{r} f_{I_{3}}<f_{I_{1}}<f_{I_{2}}<f_{I_{4}}. \end{array}$$

We know that the highest inhalation frequency component, *f*_*I*_4__, within the flow will produce the shortest inhalation period, *T*_*I*_4__. Thus, the tertiary inhalations periods are completely embedded within the lowest inhalation frequency, *f*_*I*_3__, where,


(13) $$\begin{array}{r} T_{I_{4}}<T_{I_{2}}<T_{I_{1}}<T_{I_{3}}. \end{array}$$

**Figure 3:**
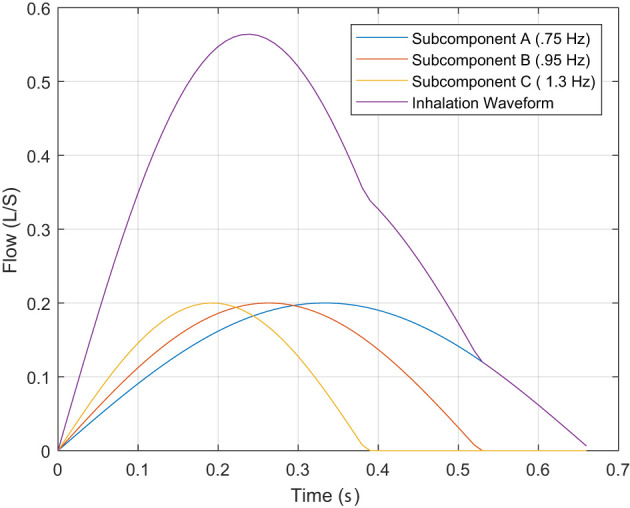
This describes the inhalation phase of breathing with its decomposed impulses associated to frequency sub-components.

This embedding of the inhalation periods, shown in Figure 3, where the inspired sub-components produce the summed output and the *T*_*I*_ discussed at the beginning of the subsection. Therefore, the characteristic of *T*_*I*_ only describes the lowest “instantaneous” frequency, causing the higher frequencies components that impact the WoB produced during inhalation (shown in Equation 5) to not be captured within this approach of characterizing breathing. This failure to capture higher frequency components that have an exponential impact on the approximation of the turbulent and viscous forces leads to an underestimation of breathing forces.

### 3.3. Decomposing Breathing Rhythm

Breathing rhythm is often associated with breathing frequency, similar to breathing rate and breathing period. However, rhythm is not frequency. Rhythm is an overlooked variable in characterizing breathing and the subject's ability to compensate within their breathing pattern. The rhythm is an informative characteristic on how a subject compensates in order to maintain alveolar ventilation by altering their *T*_*I*_ and *T*_*E*_, but maintaining the time during a full cycle's breath, *T*_*Tot*_ (Clark and Von Euler, 1972). This relationship of how *T*_*I*_ and *T*_*E*_ are altered can be described by the physiological effects of added resistance to breathing, shown by numerous studies (Cain and Otis, 1949; Zechman et al., 1957) and changes in respiratory drive (Clark and Von Euler, 1972). However, these studies do not directly distinguish the impact the variable frequency has on rhythm. The priority for any human or animal is to maintain alveolar ventilation (*V*_*A*_) defined by,


(14) $$\begin{array}{r} V_{A}=\left(V_{T}-V_{D}\right)\cdot B_{Rate} \end{array}$$

where *V*_*T*_ is the tidal volume, *V*_*D*_ is the dead space, and *B*_*Rate*_ is the breathing rate. Alveolar ventilation, described in Equation 14, can increase in two fundamental ways, through increased breathing rate or tidal volume. Likewise, alveolar ventilation can be reduced by decreasing the breathing rate or tidal volume. During breathing compensation due to the effect of a load (e.g., obstruction, breathing regulator, mask, etc.), we physiologically respond by decreasing the instantaneous airflow, otherwise known as decreasing the frequency of the inspired breath. This decrease in the frequency content of the respiratory signal decreases, in turn, the work and forces generated, Equation 5. This decrease in airflow without any compensation would drive our alveolar ventilation to decrease due to a decrease in tidal volume. Likewise, if we simply increase the breathing cycle, *T*_*Tot*_, to maintain *V*_*T*_, the breathing rate would decrease, described by


(15) $$\begin{array}{r} B_{Rate}=\frac{1}{T_{Tot}}\cdot 60. \end{array}$$

**Figure 4:**
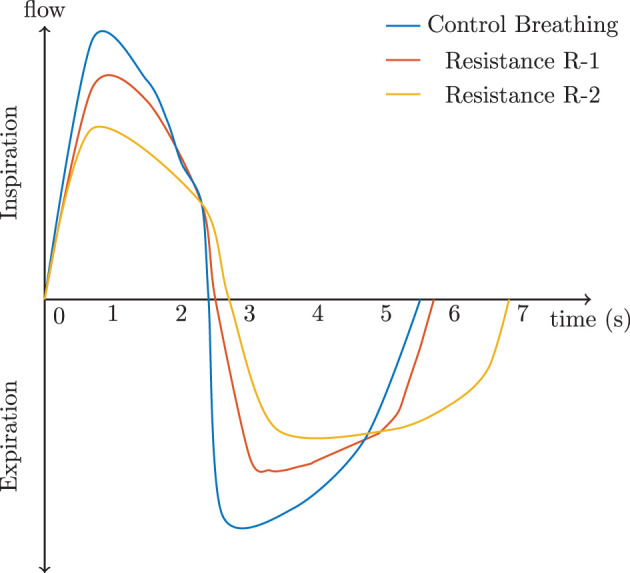
The effect of resistance on a complete respiratory cycle according to Cain and Otis (1949) and Zechman et al. (1957).

Thus, these poor rudimentary adaptions of *V*_*T*_ and *B*_*Rate*_ based on Equation 14 would drive alveolar ventilation to decrease. However, the human body adapts to the load in order to maintain alveolar ventilation by altering the within-breath rhythm. We alter the rhythm of breathing by increasing inspiration time *T*_*I*_ but also decreasing expiration time, *T*_*E*_. This allows for the respiratory system to decrease the work and maintain volume. Figure 4 shows a visualization of how airflow dynamics behave during normal breathing verse during load compensation. The most obvious reaction is how *T*_*I*_ is significantly longer and how the airflow rate is reduced. These simulated examples show the resistive load R-1 increased the time of inspiration by 2.6%, while resistive load R-2 increased it by 17%. During expiration, R-1 showed an increase of 6%, while R-2 showed a 33.5% increase.

To summarize, since breathing frequency is entirely related to the velocity pattern in Equations 3 and 4, it is also related to WoB and can impact how we consider compensation and rhythm. A significant change in *T*_*I*_ affects WoB, which is a consequence the body tries to avoid. The human body tries to obtain an optimal breathing rate that produces the required alveolar ventilation with the minimum amount of work of the respiratory muscles. For this reason, the respiratory system adjusts itself decreasing *T*_*E*_, thus, minimizing the effect on the rate of breathing. The development of improved methods that examine rhythm and its link to alveolar ventilation can lead to gaining insights on when the gas exchange between the alveoli and the external environment (CO_2_ and O_2_ ratios) cannot be maintained.

## 4. Non-sinusoidal Breathing's Impact on WoB Models

**Figure 5:**
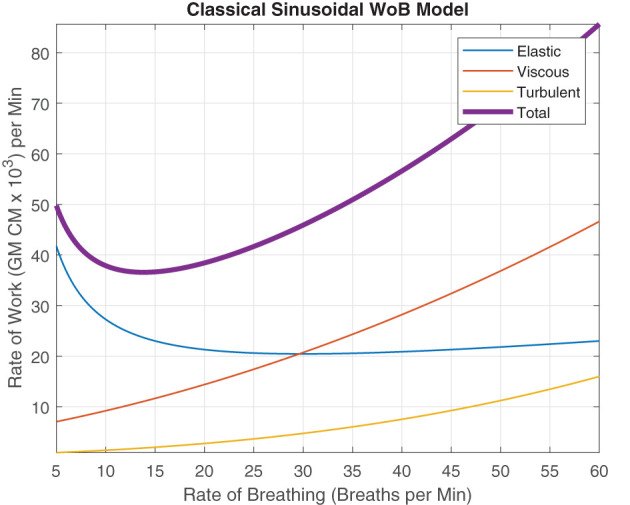
Rate of Work modeled using sinusoidal waves as Described by Otis et al. (1950), describing the relationship between elastic, viscous, turbulent and total WoB per minute, when alveolar ventilation is 6 L/min and the dead space is 200cc.

Our current breathing models and interpretation of these models are impacted through this clear delineation that breathing rate, rhythm, period, and frequency are fundamentally different because humans do not breathe sinusoidally. Through this consistent classical sinusoidal modeling, the quintessential graphical representation of WoB in Figure 5 is predominately known but neglects the non-sinusoidal breathing and the errors it might produce.

This well known classical depiction of WoB (Otis et al., 1950), assumes the x-axis as the variable “frequency,” rather than delineating the term as “rate of breathing” where we classically describe the average WoB for a sinusoidal wave over a minute by,


(16) $$\begin{array}{r} {\bar{W}}_{Tot}=\frac{1}{2}KfV_{T}^{2}+\frac{1}{4}K^{\prime }\pi^{2}{\left(fV_{T}\right)}^{2}+\frac{2}{3}K^{\prime \prime }\pi^{2}{\left(fV_{T}\right)}^{3}. \end{array}$$

This interpretation in Equation 16 and its respective graphic is only accurate when $f=\frac{1}{T_{Tot}}$ and the breathing is composed of a single frequency, creating a continuous sinusoidal waveform for a minute interval (or *f* = *B*_*Rate*_). Therefore, as the subject's instantaneous frequency of inhalation changes but maintains the same breathing rate, a large error or uncertainty regarding their “true work” of breathing occurs, making this figure an invalid representation of WoB. Therefore, this error is critical for capturing and characterizing to describe the fundamental dynamics of breathing to design life-support systems and evaluate breathing performance.

### 4.1. Frequency and Breathing Rate as Function of Work

In this modeling of WoB, we assume the inspiratory breath is only composed of a single instantaneous frequency for simplicity and the expiratory phase of the breath is passive. In addition, we maintain the same dead space (*V*_*D*_ = 200*cc*), alveolar ventilation (*V*_*A*_ = 6*L*/*min*) and mean coefficients of resistance (*K* = 8.52*cmH*_2_*O*/*L*, $K^{\prime }=3.5cmH_{2}O/\left(L/S\right)$, and $K^{\prime \prime }=1.5cmH_{2}O/{\left(L/S\right)}^{2}$) that was originally presented in the classical WoB paper using sinusoidal waves (Otis et al., 1950). However, the differences between frequency and rate for characterizing breathing dynamics are delineated by introducing *B*_*Rate*_ into Equation 5 to calculate the mean WoB, $\bar{W}$, to improve the current accepted sinusoidal model described in Equation 16 and Figure 5. We distribute *B*_*Rate*_ across Equation 5 to evaluate the impact how the frequency of the instantaneous inspiratory flow impacts WoB across the elastic, viscous, and turbulent components, shown, respectively by,


(17) $$\begin{array}{r} {\bar{W}}_{Elastic}=\frac{1}{2}B_{Rate}K{\left(\frac{V_{A}}{B_{Rate}}+V_{D}\right)}^{2} \end{array}$$

(18) $$\begin{array}{r} {\bar{W}}_{Viscous}=\frac{1}{4}K^{\prime }\pi^{2}B_{Rate}f{\left(\frac{V_{A}}{B_{Rate}}+V_{D}\right)}^{2} \end{array}$$

(19) $$\begin{array}{r} {\bar{W}}_{Turbulent}=\frac{2}{3}K^{\prime \prime }\pi^{2}B_{Rate}f^{2}{\left(\frac{V_{A}}{B_{Rate}}+V_{D}\right)}^{3}, \end{array}$$

where alveolar ventilation, *V*_*A*_ and the dead space *V*_*D*_ are accounted for by addressing the tidal volume as, $V_{T}=\frac{V_{A}}{B_{Rate}}+V_{D}$. Thus, the combination of these components of work produces the total mean work,


(20) $$\begin{array}{r} {\bar{W}}_{Tot}={\bar{W}}_{Elastic}+{\bar{W}}_{Viscous}+{\bar{W}}_{Turbulent}. \end{array}$$

### 4.2. Elastic Component of WoB

**Figure 6:**
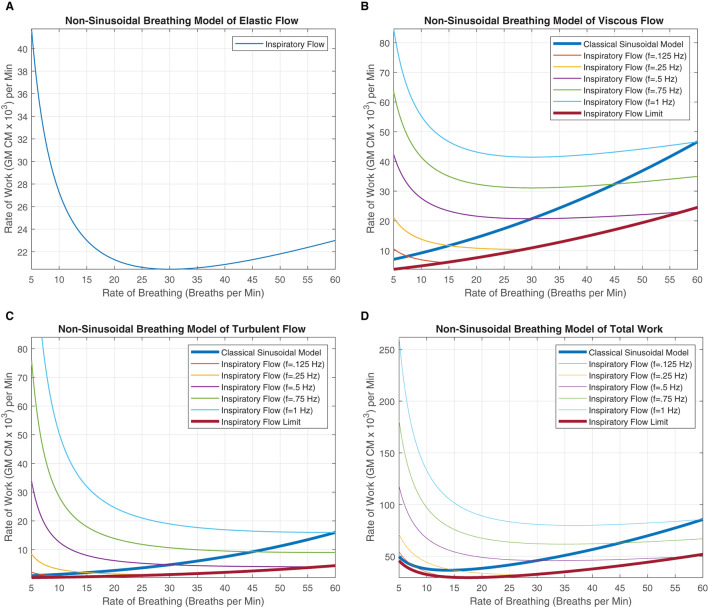
These figures were generated from Equations 18–20. Various instantaneous flow frequencies were provided and assumed to be constant over a range of breathing rates. **(A)** The classical sinusoidal WoB model was added to demonstrate the degree of the error produced when more realistic breathing patterns are introduced into the modeling framework, and it is only accurate at a single instance (The intersection between the instantaneous flow frequency curves). **(B)** The classical sinusoidal model also provides pivotal insight into state changes within the breathing patterns, where to the left of the intersection *T*_*I*_ < *T*_*E*_, to the right of the intersection *T*_*I*_ > *T*_*E*_, and at the intersection, *T*_*I*_ = *T*_*E*_ produces a true sinusoidal waveform. An instantaneous inspiration flow limit is placed with **(C,D)**, which constrains the range of the model since the instantaneous flow's *T*_*I*_ is bounded by the breathing rates *T*_*Tot*_. This flow limit was further constrained to mimic a minimum expiratory time of 10% of *T*_*Tot*_ otherwise thought of as a 90% duty cycle. The exponential decrease in work can be seen across all the instantaneous flow frequencies as the rate of breathing increases. This decrease is contributed to fixing the alveolar ventilation at 6*L* per minute, causing the human to take larger, deeper breaths since the rate is low, but the alveolar ventilation must be obtained. As the breathing rate increases, each breath's *V*_*T*_ can decrease, allowing us to model more shallow breathing within human subjects.

We can note that the elastic component of work within Equation 17 has no instantaneous flow frequency variable in the equation. The elastic work is strictly driven by the amount of air that is displaced over the time interval. The rate of breathing *B*_*Rate*_ and the amount of airflow governed by *V*_*T*_ are the determinants for the WoB within the elastic component. This delineation between instantaneous frequency of flow and rate of breathing variable was highlighted in detail in prior sections. Since Figure 5 came from the original model and their elastic function is strictly highlighting how elastic work increases as more air is displaced, this is a function of breathing rate and not the instantaneous frequency of inspiration. Therefore, this does not impact the elastic work, and there is no change within the WoB modeling for the elastic force components within the respective classical figure and non-periodic model, as seen in Figure 6A. This is identical to the classical sinusoidal model, except for the minor notation differences in which the original work referred to this variable as frequency, and we delineated this variable as the rate of breathing.

### 4.3. Viscous and Turbulent Component of WoB

In Equations 18 and 19, a clear delineation between breathing rate and instantaneous frequency is absolutely necessary for improving our quantification of the WoB. These equations are presented in Figures 6B,C, demonstrating how WoB is a function of both instantaneous frequency and rate of breathing to advance beyond sinusoidal waveforms to improve their understanding of the WoB. The error between the classical sinusoidal and non-sinusoidal models can range from zero to 10x or higher levels of work. Through the delineation between breathing rate and instantaneous frequency, we can see that the breathing rate has a greater direct impact on alveolar ventilation and dead space since the breathing rate is encapsulated within the square and cubic functions of the viscous and turbulent flows, respectively. Similar to the classical sinusoidal model, as *V*_*D*_ increases, the WoB increases. However, within the non-sinusoidal WoB model, the instantaneous frequency flow curves are altered by the breathing rate and do not follow the same trajectory relative to each other. We can note that the rate of change (the derivative) of the WoB is different across the various instantaneous frequencies curves, shown in Figures 6B,C. These different WoB rate changes are linked to how the instantaneous frequency is proportioned to the breathing rate, greatly impacting the total work produced.

As the instantaneous frequency changes within a specific breathing rate, the proportion or percentage of inspiratory time parallels similarity to a digital circuit's duty cycle. The duty cycle is expressed as a percentage of how long the load (the inspiratory breath) is “on” during a cycle (the breathing rate). This key conceptual point is expressed in detail in the section “Decomposing Breathing Period,” where *T*_*I*_ and *T*_*E*_ are not equal, and they compete with each other for the breathing rate's cycle time. Table 1, demonstrates this competition between *T*_*I*_ and *T*_*E*_ through the calculation of their duty cycle, where we calculate the instantaneous inspired frequency, $\frac{1}{2\cdot f}=T_{I}$, and breathing rate, $T_{Tot}=\frac{60}{B_{Rate}}$ to evaluate the proportional change.

**Table 1:** Duty cycle of the respiratory waveform.

**Instantaneous Frequency (Hz)**	**Breathing rate (BPM)**				
	**7.5**	**15**	**30**	**45**	**60**
0.125	50.0%	100.0%	Limit	Limit	Limit
0.25	25.0%	50.0%	100.0%	Limit	Limit
0.50	12.5%	25.0%	50.0%	66.6%	100.0%
0.75	9.38%	18.75%	37.5%	50.0%	75.0%
1.00	6.25%	12.5%	25.0%	33.3%	50.0%

***Duty Cycle Example A***. A single instantaneous inspired flow frequency of *f* =.25 Hz with a breathing rate of *B*_*Rate*_ = 15*BPM*, where *T*_*I*_ = 2 s and *T*_*Tot*_ = 4 s. The duty cycle is quantified by,


$$\begin{array}{l} \frac{T_{I}}{T_{Tot}}\cdot 100=\frac{2}{4}\cdot 100=50\%, \end{array}$$

which produces the classical sinusoidal case since the duty cycle is 50%.

***Duty Cycle Example B***. A single instantaneous inspired flow frequency of *f* = 1 Hz with a breathing rate of *B*_*Rate*_ = 7.5*BPM*, where *T*_*I*_ = 0.5 s and *T*_*Tot*_ = 8 s. The duty cycle is quantified by,


$$\begin{array}{l} \frac{T_{I}}{T_{Tot}}\cdot 100=\frac{.5}{8}\cdot 100=6.25\%. \end{array}$$

***Duty Cycle Example C***. A single instantaneous inspired flow frequency of *f* =.125 Hz with a breathing rate of *B*_*Rate*_ = 30*BPM*, where *T*_*I*_ = 4 s and *T*_*Tot*_ = 2 s. The inspiration time can not exceed the breathing period of *T*_*Tot*_ to drive the duty cycle over 100%.

### 4.4. Total WoB Model

Regarding the total WoB, we compare the classical sinusoidal model, *WoB*_*S*_, to the non-sinusoidal model, *WoB*_*NS*_, based on a range of breathing rates and instantaneous inspiratory frequencies of flow. A percent change, *P*_*C*_, between these models were applied by


(21) $$\begin{array}{r} P_{C}=100\cdot \frac{WoB_{NS}-WoB_{S}}{\left|WoB_{S}\right|}, \end{array}$$

and are shown in Table 2 to demonstrate the significant error a sinusoidal model can produce limiting our understanding of breathing dynamics. The largest errors occur within the range of 7.5–20 breaths per minute, which healthy humans typically breath around (Russo et al., 2017). Thus, when considering the average human's breathing rate of 15 breaths per minute, the individual can have extremely different levels of work with percent changes ranging from 28 to 314% across the various potential single instantaneous inhalation frequency utilized within a human's breathing repertoire. Furthermore, dominant inspiratory frequencies can go beyond 1 Hz signal, especially when multiple mixtures frequencies of instantaneous flow occur during the inspiratory phase of the breath. On the other hand, we also see an underestimation of work between the ranges of −20 to −40% when the inspiratory frequency is lower than the breathing rate.

**Table 2:** Work of breathing percent change from sinusoidal model.

**Instantaneous Frequency (Hz)**	**Breathing rate (BPM)**				
	**7.5**	**15**	**30**	**45**	**60**
0.125	0.0%	–20.0 %	Limit	Limit	Limit
0.25	28.1%	0.0%	–30.3%	Limit	Limit
0.50	101.1%	48.2%	0.0%	–25.3%	–41.2%
0.75	196.5%	107.2%	35.5%	0.0%	–21.8%
1.00	314.4%	177.2%	76.1%	28.6%	0.0%

## 5. Discussion

The analysis of differentiating flow rate, rhythm, period, and frequency illuminated the clear distinction between these variables and how they characterize the breathing dynamics differently. The instantaneous flow velocity was long sought after to mathematically define and characterize shape to explain patterns of neural stimulation of respiratory muscles (Milic-Emili and Zin, 2011), and the muscular, elastic, flow-resistive, and inertial forces of breathing (Gray and Grodin, 1951; Milic-Emili and Zin, 2011). As our analyses point out, the characterization and decomposition of the flow shape first require us to understand the term “frequency” and its true meaning within these initial models. Although these variables that characterize breathing essentially reveal different aspects of a subject's breathing and physiological state, we demonstrated that no single variable properly characterizes the shape fully to decompose the flow waveform and relate it to work or forces produced. As Milic-Emili has mentioned, no solution has ever been generated to decompose instantaneous flow into a meaningful model that enables interpretation and prediction (Gray and Grodin, 1951; Milic-Emili and Zin, 2011). However, we must first consider these breathing waveforms as non-sinusoidal waveforms that are non-stationary processes to generate such solutions to decompose these breathing waveforms, leading to clear attainable solutions. The decomposition of the flow into its appropriate frequency components, phases, and magnitudes can potentially allow us to expand these flow models to realistic interpretable, and predictive breathing mechanics. Some nascent research began to attempt to quantify the shape of instantaneous flow in the frequency domain (Abboud et al., 1986; Bachy et al., 1986; Benchetrit et al., 1989). However, the approaches were not designed for non-stationary processes. As the field of signal processing has advanced since then, a more elegant approach to frequency decomposition has arrived for biosignals that can handle non-stationary processes (Napoli et al., 2021).

**Figure 7:**
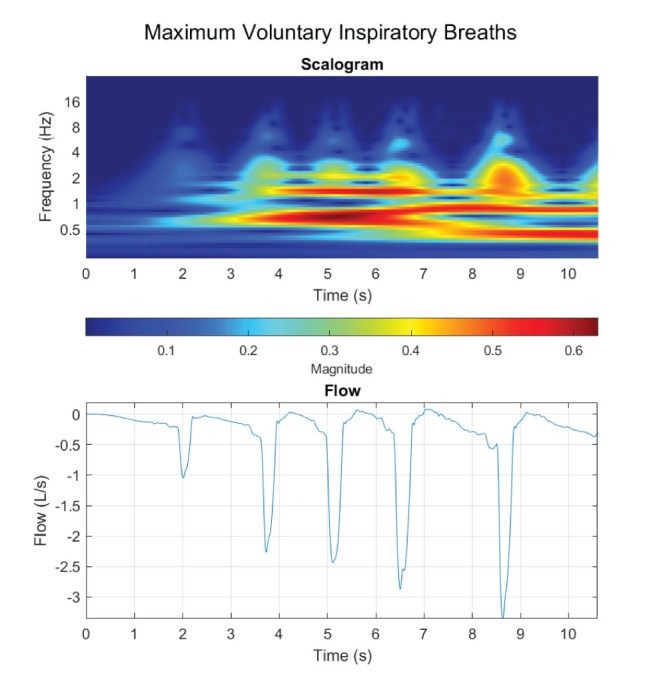
Maximum voluntary inspiratory breaths were performed, where a maximum effort was performed during the inhale phase with a relaxed controlled exhale.

Regarding the WoB model, we only presented a model in which the inspiratory flow was composed of a single frequency component and assumed the expiratory phase of the breath was passive. However, we demonstrated that *T*_*I*_ actually does not calculate the true “instantaneous” frequency, but rather we may potentially underestimate the calculation. At a minimum, a breath is composed of a linear combination of various waveforms with different frequencies, which makes up small non-linear stages of breathing. Furthermore, our Figures 6C,D for the non-sinusoidal model was constrained at only a 1 Hz instantaneous frequency, but at maximum voluntary inspiratory breaths, the instantaneous airflow frequency will extend far past 1 Hz. Figure 7 provides insight into how the flow is composed of multiple instantaneous frequencies and exceeds beyond 1 Hz by applying the continuous wavelet transform with a Morse mother wavelet.

Proper decomposition of such waveforms requires further development to evaluate a range of contributing frequency components to the inspiratory phase of the breath. Although the “fundamental” frequency of the inspired component (the component with the largest *V*_*T*_) would be the first to evaluate, Figure 6D demonstrated how other higher instantaneous frequencies could potentially be critically large contributors as a secondary or tertiary frequency component to the calculated WoB model. This is not only pivotal for calculating the work generated from breathing, but also to improve insights on how instantaneous flow should be delivered to the human on a ventilator or regulator. This model lays the foundation for modeling an inspiratory breath composed of multiple non-sinusoidal frequency components.

The non-sinusoidal model also provides some pivotal insights on breathing dynamics and can potentially describe how we many compensate during breathing loads and deterioration of breathing mechanics due to the progression of diseases, environmental conditions, and physical stresses. The classical model demonstrated that slower rates of breathing elicited a lower amount of work performed. However, due to non-periodic breathing, we simply know that is not always true. The reduction of your breathing rate does not necessarily decrease your work of breathing. Likewise, if one's breathing rate is high, it does not necessarily mean their work is high as previously modeled. The work is a function of the breathing rate and frequency of the inspiratory airflow impacting the duty cycle of the breath. As we discussed within Section 4.3 the derivative of these instantaneous frequencies curves on the WoB figure demonstrates how a person can adjust their duty cycle to maintain alveolar ventilation but reduces their work, hence load compensation. In addition, as other parameters change, like *V*_*A*_, *V*_*D*_, humidity, etc., the Pareto optimality of a human's breathing will dynamically change. Thus, the real question is, can the human compensate organically find their new optimal breathing, or at least can they be trained to find their new optimal breathing as conditions change adversely in humans. This concept will be pivotal in the future to better understand compensation during disease progression, ventilator weaning, adapting to environmental stressors, and applications of respiratory training.

## 6. Conclusion

The importance of advances in breathing mechanics is indisputable. Modeling breathing dynamics is the key to understanding the progression of many diseases, human performance, and the development of life support systems. However, we still have many unsolved problems with ventilatory weaning, prognostics, and the prediction of breathing failure as diseases progress. It raises a fundamental question, are these unsolved problems associated with us having an imprecise and inaccurate model? Even though we know we do not breathe in a sinusoidal fashion, the community continues to examine and model breathing sinusoidally (Liotti et al., 2001; Van Leeuwen et al., 2009; Matecki et al., 2016). Furthermore, due to this imprecise characterization of breathing, are specific reported results conflated, improperly lead to different hypothesizes and conclusions when it comes to compensation, changes in breathing rate/frequency, and the work of breathing? Ultimately, this prevents us from clearly delineating these impactful breathing terms to characterize breathing and improve our models to discriminate diseases and their progression, improve life support systems, and advance our understanding of human performance.

The classical WoB model demonstrated that the work performed consisted of elastic (~70%) and inelastic components (~30%), where pleural pressure is required to accurately assess work of breathing. This demands an esophageal balloon to be placed within the subject to capture the inelastic forces. However, in most settings, this is not feasible. Hence when pressure and volume measurement at the mouth are utilized, they do not capture these inelastic components contributing to the total work. Otis, Fenn, and Rahn understood this and developed instantaneous airflow models that are still utilized today to understand the inelastic component's (turbulent and viscous flow) contribution to the total work. However, this model assumes the breathing waveform to be sinusoidal, which humans do not breathe sinusoidally. We demonstrate potential errors from –41 to 314% when comparing the classical model vs. the non-sinusoidal breathing model.

This paper aimed to illuminate the critical differences of these breathing terms, clarifying their impact quantitatively on WoB, and how we begin to conceptualize new methods to advance the field to improve our understanding of breathing. We believe the presented work accomplishes this major first hurdle within the breathing community. However, additional new analytical methods need to be pursued to improve our characterization of the shape of the instantaneous flow with respect to frequency, rate, rhythm, and period.

## Data Availability Statement

The original contributions presented in the study are included in the article/supplementary material, further inquiries can be directed to the corresponding author.

## Author Contributions

NN conceived and designed the study. NN and VR performed the analysis and wrote the manuscript. PD supervised and provided insights to the scientific content of the manuscript. All authors contributed to manuscript revision, read, and approved the submitted version.

## Conflict of Interest

The authors declare that the research was conducted in the absence of any commercial or financial relationships that could be construed as a potential conflict of interest.

## Publisher's Note

All claims expressed in this article are solely those of the authors and do not necessarily represent those of their affiliated organizations, or those of the publisher, the editors and the reviewers. Any product that may be evaluated in this article, or claim that may be made by its manufacturer, is not guaranteed or endorsed by the publisher.
